# Trust in Acquaintances, Strangers and Institutions among Individuals of Different Socioeconomic Statuses during Public Health Emergencies: The Moderation of Family Structure and Policy Perception

**DOI:** 10.3390/bs14050404

**Published:** 2024-05-13

**Authors:** Xueyan Li, Xiaoli Sun, Qianqian Shao

**Affiliations:** School of Sociology, Central China Normal University, Wuhan 430079, China; sxiolo@163.com (X.S.); shaoqianqian@mails.ccnu.edu.cn (Q.S.)

**Keywords:** institution trust, acquaintance trust, stranger trust, socioeconomic status, epidemic prevention policy, COVID-19

## Abstract

Trust plays a crucial role in effectively responding to public health emergencies. Drawing on COVID-19 survey data conducted in Hubei, China, during August 2020 with a sample size of 5494, this study investigated the influence of individuals’ socioeconomic status on trust in acquaintances, strangers and institutions, and how this relationship is moderated by epidemic prevention, policy perception and family structure. The findings showed that individuals with higher socioeconomic status tend to have higher levels of trust. Those with higher income but being married demonstrate higher trust. When perceiving epidemic prevention policies as stringent, those with higher income display increased trust in acquaintances and institutions; similarly, those with lower education levels exhibit heightened trust in acquaintances and strangers. Individuals working in social organizations express higher trust in strangers; however, their trust is compromised under stringent epidemic prevention policies due to potentially heavier work burdens.

## 1. Introduction

Trust serves as an essential driver for collective action, yielding positive outcomes and contributing to societal functioning [1]. However, the uncertainty of public emergencies can erode people’s trust, alter their perceptions of others, impede collective action and cooperation, and constrain individual and collective efforts [2,3]. The COVID-19 pandemic represents the most severe global public health emergency in a century. Its suddenness and unpredictability have induced anxiety and fear among the public, which has subsequently altered social interactions and impacted public trust [4].

Prior research has established that an individual’s socioeconomic status is a crucial factor influencing their level of trust while also serving as a protective mechanism for individual trust [5]. At the onset of the COVID-19 outbreak when epidemic prevention supplies were scarce, individuals with higher socioeconomic status could acquire necessary resources such as masks, face shields and other protective equipment through their employment or international connections, whereas those with lower socioeconomic status had limited access to such resources. Nevertheless, when confronted with an extremely uncertain public health emergency like the COVID-19 pandemic, regardless of one’s socioeconomic background or personal resources available for protection against it, anxiety and fear occur. People’s personal resources alone are insufficient to cope with negative effects arising from any public health emergency; assurance provided by social institutions along with support from social relationships can offer additional protection to the general population, thereby enhancing their level of trust. Compared to normative circumstances, an emergency like this, involving widespread impact on human lives both at societal as well as individual levels, necessitates improving risk-coping abilities via institutional assurance coupled with relational support. Therefore, during such times, people tend to place their trust not only in individuals within their social networks but also in various other entities such as social groups, organizations and institution departments.

As public health emergencies are highly risky and uncertain, institutions assume the crucial responsibility of stabilizing risk and ensuring individual safety. The information and management system released by the institutions thus becomes a guideline for the public’s lives. People’s level of trust in institutions affects their cooperation with protective measures and compliance with institutional norms [3,6]. Thus, in a public health emergency, trust serves as an essential foundation for promoting epidemic prevention policy implementation and facilitating people’s cooperation in resisting epidemics. People’s perceptions of epidemic prevention policies have direct impacts on their beliefs about their health and safety as well as their trust in institutions, others and society [7]. In an emergency policy system characterized by variability and feedback loops that respond rapidly to epidemic changes, strong public trust is required to provide immediate feedback on epidemic prevention policies. Therefore, raising levels of trust while lowering intervention costs through two-way input from both state authorities and the individuals remains critical during public health emergencies.

The population’s trust in the external world is contingent upon its enhanced resilience to risks. In addition to individuals’ economic resources, social support serves as a crucial extension, and there has been an increased recognition of the significance of family in public health crises. When uncertainty and risk infiltrate people’s everyday lives and home quarantine measures keep individuals ‘together’ at home, the role of family becomes more prominent. Generally, individuals with a more stable family structure and stronger within-family support exhibit greater resistance to external risks and possess higher levels of trust. Previous studies on the relationship between family structure and trust have primarily focused on the protective effect of marriage on trust, while neglecting the association between family size and trust [8]. This paper specifically focuses on examining how both marital status and family size impact an individual’s trust while emphasizing the pivotal role played by family in fostering trust.

It is claimed that the COVID-19 pandemic eroded Chinese citizens’ confidence in the institutions and political system, with some even asserting that it shattered China’s centennial goal of achieving the ‘Chinese dream’ [9]. Concurrently, studies demonstrate that China effectively combated the COVID-19 pandemic through robust governance, stringent regulations, extensive community control and citizen participation, as well as the judicious utilization of big data and digital technology [10]. As the first group directly and severely impacted by the outbreak of COVID-19, residents of Hubei Province witnessed the entire trajectory from its emergence to containment and eventual crisis resolution. On 23 January 2020, Wuhan City, the capital of Hubei Province, implemented a strict lockdown, which was lifted on 8 April 2020. The unprecedented challenge posed by COVID-19 forced the government to confront a trade-off between a considerable number of deaths and an economic shutdown. Wuhan City became the pioneer in enforcing stringent social confinement measures as a response to COVID-19. While there is agreement on the effectiveness of such policies in decreasing COVID-19 incidence rates, they also severely constrain liberties, demolish social capital and cause economic insecurity [11]. Understanding how individuals cope with the post-lockdown aftermath and their attitudes towards institutions and unknown others is far from trivial. It is crucial to explore their level of trust in institutions and others as well as identify mechanisms that foster trust among individuals with varying socioeconomic statuses.

Since the Wuhan lockdown, many other nations and cities around the world followed suit and implemented similar policies, such as Italy (March 13), Spain (March 15), Austria (March 16), France (March 17), Denmark (March 18), the United Kingdom (March 23) and the Netherlands (March 24). A study conducted in March and April 2020 across these Western European countries revealed that the implementation of lockdown measures resulted in an increase in citizens’ trust and satisfaction with their respective government [12]. Can such a silver lining effect in trust (rally-round-the-flag effects) be observed in the population of Hubei? The answer to this question necessitates empirical evidence due to the disparity of political systems and the difference in uncertainty associated with the timing and progress of COVID-19. Such empirical evidence can inform policy-making and facilitate the implementation of strategies in response to future public health crises.

This study used the data from the General Social Survey of COVID-19 conducted in Hubei, China during August 2020. This survey was purposefully crafted to portray the social landscape during the specific period, thereby providing valuable insights for policy-making authorities. As far as we know, there are no other comparable surveys and datasets available. So far, Zhou and Guo have used these data to explore the impact of social factors on subjective distress related to COVID-19 [13]. This survey provides a distinctive opportunity to empirically examine the trust levels of individuals with diverse socioeconomic statuses, which has not been explored by any other researchers using this dataset. The unique research context of COVID-19 provided by the timing and circumstances of COVID-19 presents an opportunity for further exploration. The unique contribution of this study lies in the fact that no other researchers have used this dataset for conducting similar studies. From a risk–trust perspective, this study aims to empirically investigate trust among individuals in Hubei Province, the relationship between individual socioeconomic status and trust during the COVID-19 pandemic, as well as how this relationship is moderated by the perception of epidemic prevention policies and family structure. The unique research context of COVID-19 provided by the timing and circumstances of COVID-19 presents an opportunity for further exploration. From a risk–trust perspective, this study aims to empirically investigate trust among individuals in Hubei Province, thereby providing evidence to addressing these inquiries.

## 2. Literature Review and Research Hypothesis

This section establishes the analytical framework of this paper by theoretically elaborating on the conceptualization of trust and the mechanism of trust formation. It formulates hypotheses based on three types of indicators for trust (acquaintance trust, stranger trust, and institution trust) to explore variations in trust among individuals with different socioeconomic statuses. Furthermore, it empirically tests the moderating effects of epidemic prevention policy perception and family structure on levels of trust among individuals with different socioeconomic statuses.

### 2.1. The Conceptualization of Trust

In the face of increasing uncertainty and risk in modern society, various disciplines have emphasized the relationship between trust and social development. They conceptualized trust from different perspectives. Psychology views individual trust as a positive expectation of others’ intentions and behaviors, which involves exposing personal shortcomings and accepting possible risks [14]. Inter-organizational trust is the anticipation of specific behaviors by one organization towards another, placing itself in a vulnerable state [15]. Sociology, management, and other disciplines focus on both parties’ traits in a trusting relationship. They argue that trust is the giver’s tendency to recognize competence, honesty and the goodwill of recipients while holding positive beliefs such as approval and recognition [16].

Although these definitions vary slightly, they all include three key elements: possible risk exposure, positive expectations and the traits of both parties involved. Therefore, when studying trust issues in public health emergencies, especially those concerning the trust formation mechanism, it is important not to lose sight of these basic elements: the risk situation, the traits of parties involved and the trust formation mechanism. The following section analyzed trust during public health emergencies in terms of these elements.

### 2.2. The Risk Situation: A Risk Perspective on Public Health Emergencies

From a risk perspective, public health emergencies are primarily risky due to the increased social uncertainty that undermines people’s trust in the external world [3]. There are two main types of risks associated with public health emergencies: uncertainty regarding health emergency and uncertainty regarding individual safety. The complexity of public health emergencies like COVID-19, which involves specialized knowledge of virus transmission, causes uncertainty that individuals cannot control or effectively cope with them on their own terms. Uncertainty about individual safety affects the safety of individuals but can be mitigated by self-protection measures, to some extent. The uniqueness of a public health emergency like COVID-19 is that the power mainly resides in the its complexity, rather than an individual’s action in self-protection. In other words, individuals are coerced by COVID-19 and lose control over their own protection, and thus surrender their rights to survival to authorities and attempt to save themselves through institutions.

Institutional norms that reduce social uncertainty can increase public trust during public health emergencies [17]. Institutions implement response measures and policies to protect public safety during such emergencies, while groups like delivery workers and couriers provide necessary supplies to reduce uncertainties around individual safety. Real-time updates on emergency management released by institutions help us to improve understanding among the general population and reduce related uncertainties.

### 2.3. The Traits of Parties Involved: The Socioeconomic Status of Trusting Parties and the Plurality of Trusted Parties

#### 2.3.1. The Plurality of Trusted Parties

In public health emergencies, a “risk–trust” perspective enables us to understand how people perceive society in terms of its inherent complexity and uncertainty. The primary function of trust is to reduce social complexity by boosting the capacity for uncertainty tolerance [18]. Overwhelming social complexity and uncertainty put established trust foundation to the test. What can be relied upon during a public health emergency like COVID-19? Previous studies on trust under public emergency primarily focus on institution trust. A recent survey conducted in Sweden revealed that the COVID-19 led to an increase in the levels of trust towards both institutions and unknown others [19]. As mentioned earlier, trust in the government’s action capacity will promote compliance with epidemic prevention measures; trust in unknown individuals acting responsibly increases the likelihood of addressing collective problems such as hoarding toilet papers. Both forms of trust are crucial during public health emergencies like COVID-19.

It is acknowledged that stranger trust is interrelated to, yet distinct from, institution trust. Furthermore, there are differences in the concept of a stranger between Chinese and Western contexts. In Western culture, with an emphasis on individualism, the principle of equality serves as the foundation for defining the average person within society as a stranger. Conversely, China is a guanxi society that values human relationships. Fei Xiaotong’s concept of Chaxugeju (the Differential Mode of Association) highlights the Chinese emphasis on distinguishing between insiders and outsiders [20]. In Chinese society, whether strangers are perceived as close or distant depends on their level of interaction with individuals.

Furthermore, during public health emergencies, social interactions are significantly suppressed and constrained, particularly with limited interaction scenarios allowed during lockdown periods. Social interaction is a manifestation of interpersonal relationships, which signify the varying degrees of affinity and security between individuals [21]. Therefore, we distinguish “strangers” into those with and without interaction based on the physical presence or absence of interaction, i.e., acquaintances and strangers. In this sense, acquaintance trust refers to the level of confidence individuals possess in those with whom they have interactions beyond a specific trusted circle, whereas stranger trust is defined as individuals lacking any form of interaction or relationship with geographically distant and inaccessible persons.

In addition to interpersonal trust, institutions play different roles in public emergencies compared to normative society. In Western contexts, institutions are seen as distant state organizations, while, in China, they refer to relevant departments at all levels closely related to people’s daily lives [22]. Especially in the context of the COVID-19 pandemic, Chinese institutions have established closer ties with the population. The complexity and uncertainty surrounding public health crises render it impossible for individuals to accurately assess risks and effectively cope with them. Consequently, people organize and adjust their daily lives based on the real-time updates of information about the epidemic. Among all sources of internet information, individuals tend to place trust in ‘officially released’ or ‘authoritatively released’ information. Access to institutions’ decision-making information enhances public security beliefs, and the dissemination of such information inspires trust. Therefore, official or authoritative releases embody people’s trust in both the organization and system, constituting institution trust.

The existing literature on normal society predominantly draws upon the concept of general trust [23], while insufficiently highlighting the distinctive characteristics of Chinese society in discerning between unfamiliar and close relationships. Additionally, under public health crises, institution-provided information becomes a guiding compass in people’s daily lives and epidemic prevention efforts, reducing uncertainties associated with these crises. In light of this perspective, this paper operationalizes indicators of trust under a public health emergency into three categories, acquaintance trust, stranger trust, and institution trust, which coexist within risk situations as COVID-19.

#### 2.3.2. The Effect of an Individual’s Socioeconomic Status

The socioeconomic status of an individual has a significant impact on their propensity to trust [24]. Previous studies have operationalized socioeconomic status using various proxy variables, including education, occupation, and income. Results from normative society research indicate that individuals with higher levels of education and income are more likely to trust others, and those with higher occupational income and job stability exhibit greater levels of trust [25]. Individuals with lower socioeconomic status may have limited resources to cope with external risks and therefore exhibit less trust in the outside world. Conversely, those with higher socioeconomic status tend to possess a better understanding of societal functioning and institutional structures [26,27], as well as receive more reciprocal resources which contribute to greater levels of trust in others and society.

This explanation of trust in a normative society has two limitations for studying trust during a public health emergency. Firstly, the concept of trust used is too broad and does not reflect the fine categorization of trust in crisis. Secondly, it fails to consider the potential impact of the specific crisis context on individuals’ behavior and social lives. The implementation of lockdown measures in March and April 2020 across Western European countries increased citizens’ trust and satisfaction with their respective government [12]. Furthermore, the general population in Italy showed increased trust in others compared to pre-pandemic levels, and individuals who contracted COVID-19 displayed higher trust towards strangers [28]. Conversely, a sudden decline in social trust, reaching one of its lowest points on record, was observed in the Netherlands after the first wave of COVID-19 [29]. During the public emergency period, the distinctive social context and modes of interaction exerted an influence on levels of trust, necessitating an examination of whether the impact of socioeconomic status on various forms of trust aligns with such relationships observed in normal society. Therefore, this study proposes Hypotheses H1a-H1c as follows:

**Hypotheses** **H1a–c.** *There is a significant positive correlation between an individual’s socioeconomic status and the level of trust under a public health emergency, including acquaintance trust (H1a), stranger trust (H1b), and institution trust (H1c)*.

### 2.4. The Trust Formation Mechanism

Zuker proposed three types of trust formation: characteristics-based, process-based, and institution-based trust mechanisms [30]. This study focuses on those based on characteristics at the micro-level and institutions at the macro-level. China’s unique relational trust is primarily based on an individual’s inclination to trust [31]. The theoretical model of “relationship–trust” emphasizes the attachment between interpersonal trust and human relationships [32]. Stronger relationships provide more emotional support and help, making them more conducive to trust formation [33]. Families, as representatives of strong ties in social relationships, play a critical role in promoting trust during public health emergencies.

In a public health emergency like COVID-19, the family takes up nearly the entire scene of daily life unfolding. As a representative of strong relationships, families satisfy people’s emotional needs, relieve psychological pressure and enhance their sense of security and trust. Family has an important influence on individual trust [34], and marital status and family size represent important aspects of family structure [35]. A stable marital and family environment is crucial for individuals to enhance their security beliefs and resist external risks, which can, in turn, improve their level of trust [8]. While there are studies showing that married people tend to have higher levels of trust than those not married [36], fewer studies exist regarding the relationship between family size and trust levels. During public health crises like the COVID-19 pandemic, larger families possess stronger emotional support and higher personal security emotions, but they also require greater supplies, resulting in increased survival pressures that are prone to anxiety, negatively affecting public trust. Therefore, this study proposes the following hypotheses:

**Hypotheses** **H2a–c.** *Marital status moderates the relationship between an individual’s socioeconomic status and trust, including acquaintance trust (H2a), stranger trust (H2b) and institution trust (H2c)*.

**Hypothesis** **H3a–c.** *Family size has a moderating effect on the relationship between individual socioeconomic status and trust, including acquaintance trust (H3a), stranger trust (H3b) and institution trust (H3c)*.

Institution-based mechanisms provide trust based on social institutional norms [30], which is a macro-level mechanism for forming trust. During public health emergencies such as the COVID-19 pandemic, institutional epidemic prevention policies have a significant impact on people’s beliefs in safety and trust [13]. Institutional norms play an essential role in Chinese people’s trusting relationships [37]. The epidemic prevention policy of lockdown and quarantine introduced during COVID-19 provides practical protection measures for individuals to reduce uncertainty about personal safety. Institutional guarantees can promote public trust formation; strong feedback from the public can facilitate the smooth implementation of anti-epidemic policies. However, strictness in implementing epidemic prevention policies varies across regions, leading to differences in protection levels and security beliefs. Consequently, we propose the following hypotheses:

**Hypotheses** **H4a–c.** *Epidemic prevention policy perception has a moderating effect on the relationship between an individual’s socioeconomic status and trust, including acquaintance trust (H4a), stranger trust (H4b) and institution trust (H4c)*.

**Figure 1:**
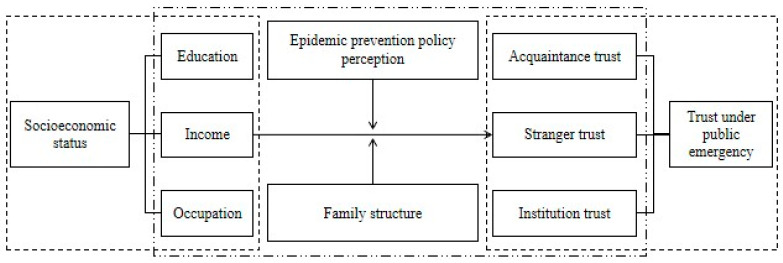
Theoretical framework.

Relational trust based on individual traits and institution trust rooted in institutional factors underpin public health emergency preparedness in Chinese society. This study identifies specific individual trait-oriented relational factors such as family structure (including family size and marital status) alongside institutional elements like epidemic prevention policy perception to better understand how these components interact to shape levels of trust during the COVID-19 epidemic. By exploring how socioeconomic status can moderate relationships between these two key drivers—namely national-level policy that offers critical safeguards against COVID-19 epidemic versus family structures that provide essential emotional support—we develop a comprehensive theoretical framework for understanding crisis response dynamics (see Figure 1).

## 3. Research Methods

### 3.1. Data Source

The data utilized in this study were obtained from the General Social Survey of COVID-19 in Hubei Province, China, which was conducted via a cross-sectional online survey in August 2020—four months after the COVID-19 epidemic in Wuhan was essentially controlled and the lockdown lifted. This survey examined various aspects of physical and mental health, family relations and life, social interaction and economic circumstances, as well as social mentality and ideology. The electronic survey was distributed through official channels such as WeChat accounts belonging to the Hubei Provincial Federation of Trade Unions, their website and an application called “Hubei Workers’ Pocket School”. A total of 25,465 individuals participated in this survey; among them, a valid sample size consisting of 5494 people took part specifically in the “Internet behavior and social mentality” module that is used for analysis herein. Further details regarding these data can be found within Zhou and Guo’s article [13].

### 3.2. Measurement of Variables

#### 3.2.1. Dependent Variable

The dependent variable in this study was trust, which was operationalized into three indicators: acquaintance trust, stranger trust and institution trust. As discussed in Section 2, the operationalization of trust in this study is characterized by a meticulous consideration of the specific context of the public health crisis of the COVID-19 pandemic and the distinctive nature of Chinese trust, with an attempt to depict people’s trust in the initial and the most severely affected regions during the COVID-19 pandemic (i.e., Hubei, China).

Acquaintance trust was assessed by measuring the level of trust individuals had in couriers or delivery workers they interacted with during the epidemic using a single question (e.g., “To what extent do you place your trust in couriers or delivery workers?”). Acquaintance trust was a type of particularized or strategic trust. Because of the unprecedented scenario created by lockdown and quarantine policies, people had significantly limited physical interactions, with the exception being when they received essential supplies from couriers and delivery workers. In a way, people’s lives depended on those couriers and delivery workers who selflessly prioritized their work over their personal health. The number of couriers and delivery workers who possessed both personal courage and authority permission to work under such severe conditions was restricted, often leading them to operate within specific regions along designated routes and therefore they interacted with the same people during the lockdown period. Consequently, this situation fostered frequent and extensive interaction, thereby aligning with the definition of acquaintance-based trust. This type of trust exemplified the unique nature of life during COVID-19 lockdown.

Stranger trust, as a type of social trust or generalized trust, was measured by evaluating the degree of trust people had in public figures, self-media influencers and the information disseminated by them through four questions (e.g., “How much do you trust self-media influencers/internet live streamers/celebrities such as singers or actors?” “How much do you trust epidemic-related information from self-media such as Weibo influencers, Toutiao influencers and WeChat official accounts?”).

Institution trust was measured by people’s trust in government announcements, information provided by official media accounts and the level of trust in government officials using seven questions (e.g., “How much do you trust epidemic-related information obtained from central official mainstream media/local official media, official announcements by central government/local government/community or village notices/announcements, and government officials/staff?”) All questions were rated on a 5-point Likert scale ranging from “very distrustful” to “very trustful”. Likert scales or ordinal variables with five or more categories can often be treated as continuous variables without compromising the validity of the planned analysis [38,39,40,41]. The scores for each indicator were averaged across relevant questions for subsequent data analysis. The analyses for ordinal variables were also conducted, treating Likert-type variables as ordinal in nature.

#### 3.2.2. Independent Variables

The independent variable in this study was individual socioeconomic status, which was primarily assessed using the three conventional variables: education, income and occupation [42]. Education level was quantified based on the duration of formal schooling in years, as follows: no education = 0; primary school = 6; secondary school = 9; high school (vocational school) = 12; university = 16; master’s degree = 19; doctoral degree = 23. Respondents were presented with 16 options, ranging from less than 1 = 1000 yuan, 2 = 1000–2000 yuan, … to 16 = above 15,000 yuan, to indicate their monthly income. Income was dichotomized into high and low categories for those with a monthly income of 5000–6000 yuan or above, with reference to the average income of 5926 yuan per month published by the Hubei Provincial Bureau of Statistics in 2020. In total, 71.3 percent of residents were in the low-income category, while 28.7 percent were in the high-income category. The low-income category was used as the reference group, and a dummy variable was created to represent the high-income category. Considering variations in the impact of the COVID-19 epidemic and job stability, occupations from the original survey (including CPC Party and government institutions; state-owned or collective enterprises; private enterprises; social organizations or village and community self-governing organizations; self-employed/self-run (partnership) enterprises; freelance work; others) were consolidated into four categories: public sector units (encompassing CPC Party and government institution and state-owned or collective enterprises); private enterprises (including private enterprises and self-employed/self-run (partnership) enterprises); social organizations (comprising social organizations or village and community self-governing organizations); other occupational categories encompassing freelance work; and others. In this study, public sector units characterized by minimal impact from the COVID-19 epidemic and strong job stability served as the reference group, while three dummy variables were created to represent private enterprises, social organizations, and other occupational categories.

#### 3.2.3. Moderating Variables

This study incorporated two moderating variables, namely epidemic prevention policy perception and family structure. The evaluation of the stringency of local lockdown measures was used to measure the public’s policy perception of epidemic prevention, with a scoring system ranging from “not strict at all” to “very strict”. Family structure was assessed based on marital status and family size. Marital statuses were categorized into two groups: in marriage (including first married and remarried) and not in marriage (including unmarried, divorced, widowed, and cohabiting). A dummy variable representing being in a marriage was created using not being in a marriage as the reference group. Family size referred to the number of economically dependent family members including the respondents of this study. Descriptive statistics for the main variables are presented in Table 1, while Table 2 displays the correlation analysis between variables. The correlation analysis revealed significant associations among all variables except for stranger trust with income.

#### 3.2.4. Control Variables

This study included four control variables, i.e., gender, age, place of residence, and political affiliation. Males constituted 61.6% of the survey participants, while females accounted for 38.4%. Overall, 77.3% resided in urban areas, whereas 22.7% in rural areas. Regarding age distribution, 65.7% fell within the range of 21 to 40 years old, with 9.4% being less than or equal to 20 years old, and 14.9% being above 40 years old. A total of 29.9% were China Communist Party members, and 71.1% were not. Those who were female, living in rural areas, and not affiliated with CPC were seen as the reference group.

### 3.3. Data Analysis Methods

This study employed OLS regression analysis to investigate the association between individuals’ socioeconomic status and trust, as well as to examine the potential moderating effects of epidemic prevention policy perception and family structure on this relationship. Additionally, an interaction model was utilized to explore how epidemic prevention policy perception and family structure moderate the link between socioeconomic status and trust. The analyses for ordinal variables were also conducted, treating Likert-type variables as ordinal in nature, to test the robustness of OLS regression for continuous variables. However, due to the violation of the assumption of parallelism (proportional odds) within the ordinal model, logit regression was deemed not appropriate in this context and could compromise the credibility of its results. Therefore, a series of multinomial regression analyses were performed instead, which yielded consistent results with OLS regression analyses, as presented in Supplementary Materials. Consequently, only OLS regression analyses for continuous variables are presented and discussed herein.

## 4. Results

### 4.1. Analysis of Factors Influencing Trust

This study employed regression analysis to examine the impact of socioeconomic status, family structure and epidemic prevention policy perception on three types of trust: acquaintance trust, stranger trust and institution trust (Table 3). Model 1 included only control variables, while Model 2 added socioeconomic status variables such as education, income and a set of dummy variables representing occupation. To reveal the influence of the COVID-19 epidemic and job stability on trust, we used public sector units as the reference group since they were less affected by the epidemic and had higher stability. Model 3 incorporated family structure and epidemic prevention policy perception.

The results from Model 2 for acquaintance trust indicate a significant positive association between education and acquaintance trust, suggesting that individuals with higher levels of education exhibit stronger trust in acquaintances. Compared to those working in public sector units, individuals with freelance work demonstrate lower levels of acquaintance trust.

Regarding Model 2 for stranger trust, the results reveal that those with higher education levels display higher trust in strangers. Those with social organization work positively influence individuals’ trust in strangers compared to those working in public sector units; however, individuals with freelance work exhibit lower levels of trust in strangers. Finally, Model 2 for institution trust indicates a significant association between institution trust and both education and income, indicating that individuals with higher levels of these two factors tend to have greater faith in institutions. Moreover, compared to individuals working in the public sector, individuals working in social organizations have a lower level of trust in institutions.

Model 3 for acquaintance trust, stranger trust and institution trust includes family structure variables and an epidemic prevention policy perception variable. Marital status exhibited a significant positive correlation with trust in both acquaintances and strangers, indicating that married individuals tended to have higher levels of trust compared to those not married. Family size and the perception of the epidemic prevention policy were found to be significantly positively associated with all three types of trust. Specifically, a larger family size was linked to higher levels of trust. Stricter perceptions regarding epidemic prevention policies were associated with increased interpersonal and institution trust. In summary, our findings partially support research hypotheses H1a-H1c regarding how different aspects related to socioeconomic status can predict various forms of trust during the COVID-19 epidemic.

### 4.2. Moderating Effects of Family Structure and Epidemic Prevention Policy Perception

Hierarchical regression analysis was employed to examine the moderating effects of family structure and epidemic prevention policy perception on trust among individuals with varying socioeconomic statuses. Model 1 included socioeconomic status variables, while Models 2–4 incorporated the moderating variables and their interaction terms in sequence.

Model 2 and 4 for acquaintance trust, presented in Table 4, indicate that marital status and epidemic prevention policy perception positively moderate acquaintance trust across income levels. This suggests that marital status and epidemic prevention policy perception strengthened the positive and significant relationship between income level and acquaintance trust. Moreover, epidemic prevention policy perception negatively moderated the relationship between education and acquaintance trust, whereas epidemic prevention policy perception had a positive moderating effect on the association between occupation (working in private sector) and acquaintance trust.

In Model 4 for stranger trust, the perception of the epidemic prevention policy negatively moderated the association between education level and stranger trust, indicating that lower levels of education were associated with higher levels of trust in strangers under such circumstances.

Model 2 of institution trust, presented in Table 4, revealed that marriage positively moderated the relationship between education level and institution trust. Specifically, marriage enhanced institution trust among individuals with a higher level of education. In Model 4, epidemic prevention policy perception positively moderated the relationship between income and institution trust. In other words, when there is a stringent perception of the epidemic prevention policy, high-income individuals tend to have increased trust in institutions. However, epidemic prevention policy perception negatively moderated the trust in institutions among those working in social organizations. Under this moderation, there was a decrease observed for those employed by social organizations compared to those working within the public sector.

In summary, our findings validate the moderating effects of marital status, family size and epidemic prevention policy perception on socioeconomic status and trust. Hypotheses H2a–H2c, H3a–H3c and H4a–H4c are partially validated. Marital status has a moderating effect on institution trust among individuals with different levels of education, and it positively moderates the trust of high-income groups in acquaintances. Perceptions of epidemic prevention policy not only have a moderating effect on trust in acquaintances and strangers among individuals with different levels of education, but also moderate trust in acquaintances and institutions based on different income levels. Additionally, epidemic prevention policy perception positively moderates trust in acquaintances among individuals working in the private sector, while negatively moderating trust in the institutions among those working in social organizations.

## 5. Discussion

### 5.1. Individual’s Socioeconomic Status Predicts Trust

Trust serves as a crucial foundation for restoring social order and facilitating the functioning of society. Socioeconomic status emerges as a significant predictor of individuals’ trust, yet the intricate relationship between each element of socioeconomic status and every type of trust has been unveiled in this study. Specifically, not all aspects of socioeconomic status exhibit significant associations with all types of trust. Notably, education level and occupation exhibit a significant correlation with all three types of trust, while income solely demonstrates a significant correlation with institution trust.

Furthermore, those employed in social organizations exhibited higher levels of trust in strangers, compared to those working in public sectors characterized by greater job stability, as well as private enterprises and freelance workers. This may be attributed to the occupational characteristics of social organization workers. Such trust extends beyond micro-level explanations based on individual and interactional behavioral traits, particularly given the high frequency of interactions with strangers required by social organization workers during the COVID-19 epidemic, as they undertook significant efforts to prevent and control epidemic spread. Individuals employed in social organizations in this study included those working in social groups and community self-governing organizations, who played an integral role within the team for “extensive community control” [10] and implemented the stringent regulations of epidemic prevention policies. And the present study provided evidence of the substantial contribution made by these individuals to Wuhan’s efforts in combating COVID-19. Professional identity within social organizations enhances both the likelihood of trusting others and being perceived as trustworthy oneself. Prior research has emphasized the links between occupational stability, job satisfaction and trust in normative society [25]. Our study contributes a novel perspective on this relationship by highlighting how occupational identity shapes interactions that facilitate or hinder the formation of trust at macro-levels.

In addition to socioeconomic status factors, family structure and epidemic prevention policy perception are significant predictors of trust levels. Individuals with larger families exhibit higher levels of trust, while married individuals have greater trust in acquaintances and strangers. During emergencies, the emotional support and assistance provided by families serve as a crucial “safe harbor” for building trust both within and outside families.

Epidemic prevention policy perception also significantly impacts people’s level of trust, especially institutional trust. The rally-round-the-flag effects observed in the Western European countries suggests that it is the severity of the crisis, rather than the government’s institutional safeguards, that affects trust in government institutions [12,19]. However, such effects took a different form in the present study. This study found that the perceptions of the epidemic prevention policy play a pivotal role in shaping individuals’ trust towards government institutions during COVID-19. These findings align with previous research on the relationship between the satisfaction of people’s needs and their trust in institutions [43]. The positive impact of perceived institutions’ implementation of epidemic prevention policies during the COVID-19 epidemic reflects the role that institutional assurance plays in fostering public trust [29,44]. Timely updates to epidemic prevention policies provide clear guidelines for navigating crises, thereby reducing negative effects associated with the uncertainty surrounding epidemics. It is unsurprising that perceived epidemic prevention policies predict levels of public trust.

### 5.2. Moderating Effects of Epidemic Prevention Policy Perception and Family Structure

The lockdown was imposed from 23 January 2020 to 8 April 2020. Following the lifting of the lockdown, a period of societal “re-adaption” occurred for one to two months, during which individuals underwent self-adjustment and adaptation processes. This study collected data after this re-adaption period. It is noteworthy that this unique time point, characterized by limited knowledge about the COVID-19 virus, may contribute to an especially significant impact of epidemic prevention policy perception on trust levels. Additionally, as previously mentioned, the absence of rally-round-the-flag effects in this study could be attributed to the timing factor, given that such effects are primarily associated with the onset of crises. The epidemic prevention policies were continuously adjusted, moving from centralized quarantine measures such as “14 + 7” and dynamic zero clearance to optimized prevention and control measures outlined in the “20 articles,” implementing Category B Disease management protocols, transitioning from strict controls towards more precise policy implementation and normalized management practices, before ultimately returning to normal. Public sense of security and trust gradually improved over time. Therefore, these findings are specific to the particular moment in time, but still can provide insight into public trust levels during similar stages of future public crises, and also offer a useful explanation for understanding how people’s trust evolves throughout different phases of crisis situations.

Marital status has a significant positive moderating effect on education and institution trust. Under the moderation of marriage, this positive and significant relationship between education and trust in institutions is strengthened. As previously noted, prior research has consistently supported the association between education level and trust [5]. However, this relationship remains inadequately accounted for in theoretical frameworks regarding the mechanisms underlying trust formation and it is not responsive to the impact of spatially constrained family life on trust during the COVID-19 epidemic. Furthermore, our findings suggest that marriage provides individuals with psychological support, emotional support and a sense of security during public health emergencies, which can extend beyond their immediate social circle to strangers, and affect trust in institutions; this effect is particularly pronounced among those with lower levels of education. The protective role played by marriage facilitates both the restoration and recreation of trust.

Epidemic prevention policy perception not only had a direct positive impact on people’s trust, but also moderated the effect of socioeconomic status on trust. For individuals with a lower education, strict policy perception was associated with a higher level of stranger and acquaintance trust. For those with a higher income, stringent policy perception was associated with higher trust in acquaintances and institutions. Strict policy perception was linked to higher levels of acquaintance trust among those working in private enterprises. A previous study showed that strict interventions can safeguard public trust during the early stages of the COVID-19 epidemic [6], which aligns with our research findings. However, we also observed a negative impact of stronger policy perception on the trust levels of social organization workers in institutions. This can be attributed to their unprecedented and heavily burdened frontline involvement in epidemic prevention efforts, which leads to heightened work pressure, anxiety, as well as physical and psychological exhaustion, which ultimately undermine their level of trust.

Moreover, it is crucial to acknowledge that during the COVID-19 epidemic, epidemic prevention policies were directly linked to people’s safety and well-being. As such, these policies not only served as institutional safeguards to promote trust, but also bolstered the confidence of individuals, particularly those from low socioeconomic backgrounds, by ensuring their safety and enabling them to resume their daily routines. The public’s perception of these policies was closely tied to the severity of the epidemic; therefore, appropriate prevention and control measures were highly effective in enhancing trust among employees in private enterprises. However, an excessive emphasis on epidemic prevention policies could undermine trust among those working in social organizations due to the unnecessary extra burden. Thus, timely adjustments to these policies were essential for maintaining or even increasing public trust levels. Post-pandemic policy revisions aimed at safeguarding people’s livelihoods and meeting basic needs pose a significant challenge for institutions’ decision-making processes and represent a critical means of preserving public trust.

## 6. Conclusions

This study investigates the association between individuals’ socioeconomic status and trust, as well as how this relationship is moderated by epidemic prevention policy perception and family structure. The findings indicate that individuals with higher socioeconomic status exhibit greater levels of trust during times of epidemic crisis like COVID-19. Given the positive role of trust in maintaining social order and functioning, it is crucial to focus on enhancing trust among individuals with lower socioeconomic status through diverse measures, thereby reconstructing trust in the post-epidemic era. Moreover, the implementation of epidemic prevention policies in China not only directly influences trust but also moderates the impact of socioeconomic status on trust. This study reveals that perceptions of epidemic prevention policies can be a double-edged sword; while they can enhance people’s sense of security and subsequently their level of trust, they may also erode their trust when perceptions are extreme (either strong or weak). Therefore, attention should be given to those executing epidemic policies and therefore bearing an enormous burden during public health emergencies. The strong level of trust observed among individuals working in social organizations highlights their significant role during such crises due to their professional characteristics. Simultaneously, there is a need for the mutual construction of trust between social organization workers and the public to ensure the smooth execution of various policies pertaining to people’s daily lives during home isolation.

However, there are several limitations to this study. Firstly, the utilization of cross-sectional data restricts the ability to compare the relationship between socioeconomic status and public trust during a public health emergency and normal period. Secondly, the study solely relies on reported data from individuals in Hubei Province during the COVID-19 pandemic, lacking relevant information from other regions or different time periods. Future research should focus on investigating changes in public trust during crises and conducting longitudinal studies to better understand variations in public trust between normal periods and times of emergencies, thereby exploring the impact of social emergencies on public trust.

## Figures and Tables

**Table 1:** Descriptive statistics of the main variables (N = 5494).

Variables	Values	Sample Size	Percentage (%)
Gender	Male	3383	61.6
	Female	2111	38.4
Age	≤20	519	9.4
	21–30	2335	42.5
	31–40	1819	33.1
	41–50	649	11.8
	51–60	146	2.7
	>60	26	0.5
Place of residence	Urban	4247	77.3
	Rural	1247	22.7
Political affiliation	Communist Party member	1645	29.9
	Non-Communist Party member	3849	70.1
Education	Elementary school and below	203	3.7
	Middle school	399	7.3
	High school (vocational school)	1375	25.0
	Junior colleges	1270	23.1
	University and above	2247	40.9
Occupation	Public sector unit	2352	42.8
	Private enterprise	2114	38.5
	Social organization	545	9.9
	Others	483	8.8
Marital status	Not Married	2256	41.1
	Married	3238	58.9
Family size	Family members	5494	3.69 ^#^
Policy perception	Not strict at all	245	4.5
	Not too strict	263	4.8
	Fair	517	9.4
	Fairly strict	1668	30.4
	Very strict	2801	51.0

Note: # represents mean values.

**Table 2:** Correlation analysis of variables.

	Education	Income	Family Size	Policy Perception	Acquaintance Trust	Stranger Trust	Institution Trust
Education	1.000						
Income	0.334 ***	1.000					
Family size	0.029 *	0.081 ***	1.000				
Policy perception	0.182 ***	0.132 ***	0.045 ***	1.000			
Acquaintance trust	0.076 ***	0.049 ***	0.048 ***	0.165 ***	1.000		
Stranger trust	0.029 *	0.005	0.063 ***	0.119 ***	0.683 ***	1.000	
Institution trust	0.158 ***	0.099 ***	0.049 ***	0.375 ***	0.466 ***	0.486 ***	1.000

Note: * *p* < 0.05, *** *p* < 0.001.

**Table 3:** Regressions of trust.

	Acquaintance Trust			Stranger Trust			Institution Trust		
	Model 1	Model 2	Model 3	Model 1	Model 2	Model 3	Model 1	Model 2	Model 3
**Control variables**									
Gender (female = 0)	−0.048 ***	−0.050 ***	−0.041 **	−0.059 ***	−0.059 ***	−0.051 ***	−0.044 **	−0.047 **	−0.035 **
Age	0.081 ***	0.076 ***	0.041 **	−0.009	−0.010	−0.039 **	0.136 ***	0.127 ***	0.079 ***
Place of residence (rural = 0)	0.020	0.001	0.004	−0.006	−0.019	−0.016	0.024	0.010	−0.004
Political affiliation	0.019	0.002	0.010	−0.003	−0.013	−0.006	−0.012	−0.037 *	−0.016
**Independent variables (socioeconomic status)**									
Education		0.059 ***	0.032 *		0.032 *	0.008		0.141 ***	0.082 ***
Income (lower = 0)		0.024	0.004		−0.004	−0.022		0.057 ***	0.021
Occupation (public sector unit)									
Private enterprise		−0.018	−0.026		−0.023	−0.030		0.019	0.004
Social organization		0.023	0.026		0.051 ***	0.052 ***		−0.043 **	−0.033 *
Others		−0.032 *	−0.034 *		−0.037 *	−0.040 **		−0.001	−0.015
**Other variables**									
Family structure									
Marital status (not married = 0)			0.046 **			0.035 *			0.008
Family size			0.034 *			0.055 ***			0.029 *
Policy perception			0.152 ***			0.125 ***			0.341 ***
**F test**	15.138 ***	10.779 ***	20.011 ***	4.592 **	5.491 ***	12.734 ***	29.689 ***	30.399 ***	81.084 ***

Note: (1) * *p* < 0.05, ** *p* < 0.01, *** *p* < 0.001; (2) the reference group for political affiliation is non-communist party member, the reference group for occupation is the public sector unit, and the reference group for marital status is not in a marriage.

**Table 4:** Regression results of moderating effects.

	Acquaintance Trust				Stranger Trust				Institution Trust			
	Model 1	Model 2	Model 3	Model 4	Model 1	Model 2	Model 3	Model 4	Model 1	Model 2	Model 3	Model 4
**Control variables**												
Gender (female = 0)	−0.050 **	−0.048 ***	−0.048 **	−0.045 ***	−0.059 ***	−0.057 **	−0.058 ***	−0.055 ***	−0.047 **	−0.047 **	−0.046 **	−0.036 **
Age	0.076 ***	0.061 ***	0.075 ***	0.055 ***	−0.010	−0.021	−0.011	−0.029 *	0.127 ***	0.127 ***	0.126 ***	0.082 ***
Place of residence (rural = 0)	0.001	−0.001	0.001	0.006	−0.019	−0.020	−0.018	−0.014	−0.010	−0.012	−0.009	−0.005
Political affiliation	0.002	0.001	0.002	0.015	−0.013	−0.013	−0.014	0.001	−0.037 *	−0.040 **	−0.035 **	−0.016
**Independent variables (socioeconomic status)**												
Education	0.059 ***	0.071 ***	0.059 ***	0.025	0.032 *	0.042 *	0.032 *	−0.004	0.141 ***	0.118 ***	0.0137 ***	0.084 ***
Income (lower = 0)	0.024	−0.013	0.021	0.008	−0.004	−0.019	−0.009	−0.014	0.057 ***	0.036	0.054 ***	0.018
Occupation (public sector unit)												
Private enterprise	−0.018	−0.026	−0.020	−0.027	−0.023	−0.041	−0.025	−0.031 *	0.019	0.030	0.017	0.006
Social organization	0.023	0.046 *	0.022	0.026	0.051 ***	0.050 *	0.048 **	0.054 ***	−0.043 **	−0.027	−0.045 **	−0.035 *
Others	−0.032 *	−0.019	−0.032 *	−0.039 **	−0.037 *	−0.022	−0.037 *	−0.046 **	−0.001	0.013	−0.002	−0.014
**Moderating variables**												
Family structure												
In a marriage		0.032				0.023				0.006		
In a marriage×education		−0.024				−0.019				0.042 *		
In a marriage × income		0.049 *				0.018				0.025		
In a marriage × private enterprise		0.011				0.026				−0.011		
In a marriage × social organization		−0.031				−0.001				−0.017		
In a marriage × others		−0.016				−0.022				−0.019		
Family size			0.034				0.048				0.069 **	
Family size × education			0.011				0.012				−0.020	
Family size × income			−0.007				0.010				−0.030	
Family size × private enterprise			0.027				0.011				−0.013	
Family size × social organization			−0.002				0.007				−0.019	
Family size × others			−0.002				0.003				−0.006	
Policy perception				0.094 ***				0.086 ***				0.335 ***
Policy perception × education				−0.043 **				−0.057 ***				0.002
Policy perception × income				0.036 *				0.012				0.033 *
Policy perception × private enterprise				0.056 ***				0.030				0.005
Policy perception × social organization				0.002				0.018				−0.034 *
Policy perception × others				−0.006				−0.004				−0.001
**F test**	10.779 ***	7.667 ***	7.366 ***	16.468 **	5.491 ***	3.985 ***	4.714 ***	9.794 ***	30.399 **	18.985 **	19.244 **	65.376 ***

Note: (1) * *p* < 0.05, ** *p* < 0.01, *** *p* < 0.001; (2) models two, three and four incorporate the moderating variables and their interaction terms in turn; (3) the reference group for political affiliation is the non-communist party member, the reference group for income is the lower income group, the reference group for occupation is the public sector unit, and the reference group for marital status is not in a marriage.

## Data Availability

The data presented in this study are available on request from the corresponding author. The data are not publicly available due to privacy restrictions.

## Supplementary Materials

The following supporting information can be downloaded at: https://www.mdpi.com/article/10.3390/bs14050404/s1, Table S1: Multinomial regression of acquaintance trust; Table S2: Multinomial regression of stranger trust; Table S3: Multinomial regression of institution trust; Table S4-1: Multinomial regression results of moderating effects for acquaintance trust; Table S4-2: Multinomial regression results of moderating effects for acquaintance trust; Table S4-3: Multinomial regression results of moderating effects for acquaintance trust; Table S5-1: Multinomial regression results of moderating effects for stranger trust; Table S5-2: Multinomial regression results of moderating effects for stranger trust; Table S5-3: Multinomial regression results of moderating effects for stranger trust; Table S6-1: Multinomial regression results of moderating effects for institution trust; Table S6-2: Multinomial regression results of moderating effects for institution trust; Table S6-3: Multinomial regression results of moderating effects for institution trust.
