# Supplementary material for: Trust in Acquaintances, Strangers and Institutions among Individuals of Different Socioeconomic Statuses during Public Health Emergencies: The Moderation of Family Structure and Policy Perception
Source: Behav Sci (Basel). 2024 May 13;14(5):404. doi: 10.3390/bs14050404 (PMC11118019; doi:10.3390/bs14050404)
Supplement: Supplementary file 1 [file behavsci-14-00404-s001.zip › behavsci-2907058-supplementary.pdf]

Supplementary Materials

In general, the results of multinomial regression are largely consistent with those of OLS regressions. The only exceptions were the moderating coefficients of epidemic prevention policy perception between education and trust in acquaintance and stranger, and between working in social organization and trust in institution. These coefficients lost significance. However, these results do not alter the main findings and conclusions of the present study. In other words, the multinomial regression analyses yielded consistent results with OLS regression analyses, and in the present study, the results remain robustness regardless of whether the Likert-type dependent variables were treated as continuous or ordinal.

Table S1: Multinomial regression of acquaintance trust

Acquaintance trust								
Trust level	1		2		3		4	
	B	Exp(B)	B	Exp(B)	B	Exp(B)	B	Exp(B)
Gender (female=0)	0.440**	1.553	0.301*	1.351	0.175*	1.192	0.156	1.169
Age	-0.253**	0.776	-0.077	0.926	-0.025	0.975	0.037	1.038
Place of residence (rural=0)	0.084	1.088	-0.149	0.861	-0.025	0.975	0.044	1.045
Political affiliation	-0.098	0.906	0.099	1.104	-0.253**	0.777	-0.212*	0.809
Education	-0.037	0.964	-0.024	0.977	0.030*	1.031	0.038**	1.039
Income (lower=0)	0.086	1.090	-0.094	0.910	-0.014	0.986	0.079	1.082
Occupation (public sector unit)								
Private enterprise	-0.198	0.820	0.212	1.236	0.342***	1.408	0.128	1.137
Social organization	-0.403	0.669	-0.200	0.819	-0.155	0.856	-0.102	0.903
Others	-0.164	1,179	0.474	1.607	0.508**	1.662	0.222	1.249
Marital status (not married=0)	-0.201	0.818	0.020	1.020	-0.209*	0.811	0.212*	1.236
Family size	-0.034	0.966	-0.056	0.946	-0.089***	0.915	-0.057*	0.945
Policy perception	-0.572***	0.564	-0.445***	0.641	-0,256***	0.774	-0.165***	0.848
Intercept	1.870***		1.101**		1.800***		0.490	

Table S2: Multinomial regression of stranger trust

Stranger trust								
Trust level	1		2		3		4	
	B	Exp(B)	B	Exp(B)	B	Exp(B)	B	Exp(B)
Gender (female=0)	0.534*	1.705	0.255	1.290	0.138	1.148	0.198	1.219
Age	0.057	1.059	0.124	1.132	0.122	1.130	0.060	1.062
Place of residence (rural=0)	0.262	1.300	0.298	1.347	0.097	1.101	0.196	1.216
Political affiliation	0.055	1.056	0.023	1.023	0.063	1.065	0.064	1.067
Education	-0.024	0.977	0.025	1.025	0.031	1.031	0.009	1.009
Income (lower=0)	0.345	1.412	0.091	1.095	0.122	1.129	0.064	1.066
Occupation (public sector unit)								
Private enterprise	0.084	1.088	-0.748*	0.473	-0.213	0.809	-0.258	0.773
Social organization	-0.586	0.556	0.906**	2.475	1.018***	2.767	0.713*	2.041
Others	0.825*	2.281	0.145	1.156	0.444**	1.558	0.160	1.173
Marital status (not married=0)	0.003	1.003	-0.057	0.945	-0.313*	0.731	0.175	1.192
Family size	-0.117	0.889	-0.148**	0.862	-0.113**	0.893	-0.043	0.958
Policy perception	-0.945***	0.389	-0.652***	0.521	-0.607***	0.545	-0.579***	0.560
Intercept	3.065***		1.628*		3.003***		2.197***	

Table S3: Multinomial regression of institution trust

Institution trust								
Trust level	1		2		3		4	
	B	Exp(B)	B	Exp(B)	B	Exp(B)	B	Exp(B)
Gender (female=0)	-0.258	0.773	0.891*	2.438	0.358*	1.430	-0.029	0.972
Age	-0.451	0.637	-0.645**	0.525	-0.250**	0.779	-0.110	0.896
Place of residence (rural=0)	0.381	1.464	0.846	2.331	-0.054	0.947	-0.288	0.750
Political affiliation	-0.322	0.725	-0.112	0.894	0.065	1.067	0.005	1.005
Education	-0.086**	0.917	-0.031	0.970	-0.038	0.963	-0.022	0.979
Income (lower=0)	-1.032	0.356	0.378	1.460	-0.051	0.951	-0.239	0.788
Occupation (public sector unit)								
Private enterprise	-0.329	0.720	0.318	1.374	0.312	1.367	0.037	1.038
Social organization	0.342	1.408	-0.433	0.649	0.595*	1.814	0.215	1.239
Others	-0.142	0.868	0.639	1.895	0.172	1.188	-0.294	0.746
Marital status (not married=0)	1.260*	3.526	0.944*	2.571	-0.279	0.757	0.100	1.105
Family size	-0.283	0.753	-0.115	0.892	-0.152**	0.859	-0.113*	0.893
Policy perception	-1.240***	0.290	-1.646***	0.193	-1.378***	0.252	-1.119***	0.327
Intercept	4.655***		4.961***		6.877***		5.592***	

Table S4-1: Multinomial regression results of moderating effects for acquaintance trust

Acquaintance trust-1								
Trust level	1		2		3		4	
	B	Exp(B)	B	Exp(B)	B	Exp(B)	B	Exp(B)
Gender (female=0)	0.484**	1.622	0.335*	1.398	0.195*	1.215	0.168*	1.182
Age	-0.354***	0.702	-0.141	0.869	-0.056	0.946	0.022	1.023
Place of residence (rural=0)	0.142	1.153	-0.113	0.893	-0.009	0.991	0.051	1.052
Political affiliation	-0.026	0.974	0.155	1.168	-0.235**	0.791	-0.211*	0.810
Education	-0.126***	0.882	-0.087***	0.917	-0.016	0.984	-0.013	0.987
Income (lower=0)	0.299	1.348	-0.020	0.980	0.104	1.110	0.061	1.063
Occupation (public sector unit)								
Private enterprise	-0.166	0.847	0.315	1.370	0.555***	1.742	0.419**	1.520
Social organization	-0.566	0.568	-0.584	0.558	-0.157	0.854	-0.072	0.930
Others	-0.153	0.858	0.378	1.459	0.520*	1.682	0.321	1.378
Marital status (not married=0)								
In a marriage×education	0.122**	1.130	0.071*	1.074	0.061**	1.063	0.077**	1.080
In a marriage×income	-2.363***	0.290	-1.170	0.310	-1.171**	0.310	-0.759	0.496
In a marriage×private enterprise	-0.094	0.911	-0.237	0.789	-0.386*	0.680	-0.467*	0.627
In a marriage×social organization	0.466	1.593	0.686	1.985	0.039	1.039	-0.003	0.997
In a marriage×others	0.596	1.815	0.065	1.067	-0.036	0.965	-0.177	0.838
Intercept	0.727		0.009		0.982***		0.212	

Table S4-2: Multinomial regression results of moderating effects for acquaintance trust

Acquaintance trust-2								
Trust level	1		2		3		4	
	B	Exp(B)	B	Exp(B)	B	Exp(B)	B	Exp(B)
Gender (female=0)	0.486**	1.626	0.321*	1.379	0.197*	1.218	0.145	1.156
Age	-0.369***	0.691	-0.136	0.873	-0.092*	0.913	0.054	1.056
Place of residence (rural=0)	0.142	1.152	-0.104	0.901	-0.020	0.980	0.062	1.064
Political affiliation	0.009	1.009	0.174	1.190	-0.233**	0.792	-0.185*	0.832
Education	-0.047	0.954	-0.044	0.957	-0.002	0.998	0.020	1.020
Income (lower=0)	-0.570	0.566	-0.142	0.868	0.109	1.116	0.056	1.058
Occupation (public sector unit)								
Private enterprise	0.144	1.155	0.540	1.716	0.663**	1.941	0.493*	1.637
Social organization	-0.805	0.447	0.160	1.173	0.221	1.248	0.345	1.412
Others	-0.041	0.959	0.204	1.226	0.265	1.304	-0.172	0.842
Family size								
Family size×education	0.127	1.135	-0.002	0.992	0.006	0.842	0.003	0.953
Family size×income	-0.002	0.998	-0.008	1.007	-0.172	0.884	-0.048	0.954
Family size×private enterprise	-0.108	0.898	-0.101	0.904	-0.088	0.915	-0.100	0.905
Family size×social organization	0.121	1.129	-0.079	0.924	-0.091	0.913	-0.112	0.894
Family size×others	0.023	1.023	0.049	1.050	0.065	1.068	0.094	1.098
Intercept	0.067		-0.464		1.046**		-0.064	

Table S4-3: Multinomial regression results of moderating effects for acquaintance trust

Acquaintance trust-3								
Trust level	1		2		3		4	
	B	Exp(B)	B	Exp(B)	B	Exp(B)	B	Exp(B)
Gender (female=0)	0.454**	1.575	0.304*	1.355	0.194*	1.214	0.146	1.157
Age	-0.290**	0.748	-0.073	0.929	-0.064	0.938	0.072	1.074
Place of residence (rural=0)	0.068	1.071	-0.131	0.877	-0.018	0.982	0.063	1.065
Political affiliation	-0.099	0.906	0.093	1.097	-0.264**	0.768	-0.202*	0.817
Education	-0.039	0.962	-0.023	0.977	0.044	1.045	0.057	1.059
Income (lower=0)	1.000	2.718	1.050	2.857	0.343	1.409	0.291	1.338
Occupation (public sector unit)								
Private enterprise	1.663**	5.274	2.128***	0.839	2.059***	7.839	0.773	2.167
Social organization	-0.336	0.715	-0.606	0.546	0.048	1.049	-0.545	0.580
Others	0.711	2.037	-0.338	0.713	0.029	1.029	-0.315	0.730
Policy perception								
Policy perception×education	0.001	1.000	-0.001	0.999	-0.004	0.852	-0.005	0.871
Policy perception×income	-0.615**	0.541	-0.556*	0.573	-0.161	0.927	-0.138	0.912
Policy perception×private enterprise	-0.451**	0.637	-0.457***	0.633	-0.395***	0.674	-0.142	0.867
Policy perception×social organization	-0.023	0.977	0.106	1.112	-0.055	0.947	0.105	1.111
Policy perception×others	-0.169	0.844	0.187	1.205	0.115	1.121	0.117	1.125
Intercept	1.053		0.242		0.749		0.021	

Table S5-1: Multinomial regression results of moderating effects for stranger trust

Stranger trust-1								
Trust level	1		2		3		4	
	B	Exp(B)	B	Exp(B)	B	Exp(B)	B	Exp(B)
Gender (female=0)	0.586**	1.797	0.430*	1.538	0.165	1.180	0.130	1.139
Age	-0.093	0.911	0.344**	1.411	0.052	1.153	0.010	1.010
Place of residence (rural=0)	0.297	1.345	0.193	1.213	0.104	1.104	0.104	1.109
Political affiliation	0.152	1.164	-0.080	0.923	0.104	1.109	0.158	1.171
Education	-0.158***	0.854	-0.010	0.990	-0.043	0.958	-0.004	0.996
Income (lower=0)	0.463	1.590	0.284	1.329	0.299	1.349	0.043	1.044
Occupation (public sector unit)								
Private enterprise	-0.490	0.613	0.072	1.075	0.276	1.318	0.283	1.326
Social organization	0.504	1.655	1.598***	4.945	1.063**	2.896	0.065	1.067
Others	-0.096	0.908	0.090	0.900	0.752*	2.122	-0.050	0.952
Marital status (not married=0)								
In a marriage×education	0.173**	1.189	0.102	1.107	0.081*	1.084	0.059	1.061
In a marriage×income	-2.857**	0.057	-1.610	0.392	-1.530*	0.217	-0.933	0.393
In a marriage×private enterprise	0.183	1.201	-0.551	0.577	-0.597*	0.550	-0.115	0.892
In a marriage×social organization	0.059	1.061	-0.649	0.522	-0.742	0.476	0.075	1.078
In a marriage×others	0.345	1.413	-1.98	0.333	-0.178	0.837	0.462	1.587
Intercept	0.798		2.216**		0.920*		0.155	

Table S5-2: Multinomial regression results of moderating effects for stranger trust

Stranger trust-2								
Trust level	1		2		3		4	
	B	Exp(B)	B	Exp(B)	B	Exp(B)	B	Exp(B)
Gender (female=0)	0.589**	1.803	0.170	1.186	0.167	1.182	-0.150	0.861
Age	-0.076	0.927	0.130	1.138	0.002	1.002	0.216*	1.242
Place of residence (rural=0)	0.308	1.361	0.174	1.190	0.073	1.076	0.284	1.328
Political affiliation	0.216	1.242	0.241	1.272	0.102	1.107	0.127	1.135
Education	-0.078	0.925	0.036	1.037	-0.059	0.943	0.010	1.010
Income (lower=0)	0.767	2.153	0.128	1.136	0.039	1.039	0.706	2.025
Occupation (public sector unit)								
Private enterprise	0.003	1.003	0.310	1.363	0.983*	2.674	0.664	1.943
Social organization	-0.251	0.778	-0.698	0.498	0.657	1.930	0.771	2.161
Others	-0.726	0.484	-1.720	0.179	0.383	1.467	-0.019	0.981
Family size								
Family size×education	0.001	0.999	-0.014	0.986	0.018	1.019	0.003	1.003
Family size×income	-0.274	0.760	0.039	0.975	-0.311	0.733	-0.049	0.952
Family size×private enterprise	0.006	1.006	0.025	1.026	-0.150	0.861	-0.108	0.897
Family size×social organization	-0.042	0.959	0.053	1.054	-0.205	0.815	-0.143	0.867
Family size×others	0.371	1.449	0.503	1.654	0.170	1.185	0.200	1.222
Intercept	0.091		1.120		1.487*		2.176*	

Table S5-3: Multinomial regression results of moderating effects for stranger trust

Stranger trust-3								
Trust level	1		2		3		4	
	B	Exp(B)	B	Exp(B)	B	Exp(B)	B	Exp(B)
Gender (female=0)	0.544*	1.722	0.432*	1.540	0.036	1.037	-0.160	0.853
Age	0.052	1.054	0.390***	1.477	0.172*	1.188	0.247*	1.280
Place of residence (rural=0)	0.274	1.316	0.216	1.242	0.133	1.142	0.287	1.333
Political affiliation	0.060	1.062	-0.109	0.897	-0.020	0.980	0.101	1.107
Education	0.030	1.031	0.102	1.108	0.055	1.057	0.098	1.103
Income (lower=0)	0.115	1.122	-2.526	0.080	-0.246	0.782	0.507	1.661
Occupation (public sector unit)								
Private enterprise	-1.225	0.294	-0.921	0.398	-0.192	0.825	-1.570	0.208
Social organization	6.426*	61.716	6.213	49.176	6.065*	43.488	5.027	15.526
Others	0.501	1.651	2.035	7.651	0.879	2.409	-1.150	0.317
Policy perception								
Policy perception×education	-0.012	0.988	-0.007	0.993	-0.006	0.994	-0.014	0.987
Policy perception×income	-0.722	0.386	0.134	1.143	-0.333	0.716	-0.379	0.685
Policy perception×private enterprise	-0.083	0.920	-0.325	0.723	-0.117	0.889	0.327	1.386
Policy perception×social organization	0.215	1.239	0.137	1.147	-0.049	0.952	0.407	1.502
Policy perception×others	-1.179	0.308	-1.052	0.349	-1.113	0.329	-0.883	0.413
Intercept	1.741		1.432		1.342		0.810	

Table S6-1: Multinomial regression results of moderating effects for institution trust

Institution trust-1								
Trust level	1		2		3		4	
	B	Exp(B)	B	Exp(B)	B	Exp(B)	B	Exp(B)
Gender (female=0)	0.356	1.427	0.720*	2.055	0.351*	1.420	-0.006	0.994
Age	-0.435**	0.647	-0.075	0.927	0.041	1.042	0.009	1.009
Place of residence (rural=0)	0.315	1.371	0.672	1.959	0.103	1.109	0.226	1.254
Political affiliation	0.408	1.504	-0.185	0.831	-0.103	0.902	0.390*	1.477
Education	-0.160***	0.852	0.092	0.857	0.004	1.004	0.020	1.020
Income (lower=0)	0.012	1.012	0.019	1.020	-0.054	0.948	0.410	1.507
Occupation (public sector unit)								
Private enterprise	-0.481	0.618	0.073	1.075	0.283	1.327	-0.200	0.819
Social organization	-0.349	0.705	1.397	4.043	0.548	1.731	-0.968	0.380
Others	-0.388	0.678	0.832	2.299	0.417	1.518	0.332	1.394
Marital status (not married=0)								
In a marriage×education	0.047	1.048	-0.154	0.857	0.072*	0.926	-0.032	0.968
In a marriage×income	-1.545	0.213	2.739	15.473	-0.191	0.826	0.459	1.583
In a marriage×private enterprise	0.186	1.205	-0.677	0.508	-0.346	0.707	-0.493	0.611
In a marriage×social organization	0.596	1.815	-0.181	0.835	-0.508	0.602	-0.016	0.984
In a marriage×others	1.352	3.865	-0.856	0.425	-0.456	0.634	0.471	1.602
Intercept	1.118		5.390***		1.365*		2.234**	

Table S6-2: Multinomial regression results of moderating effects for institution trust

Institution trust-2								
Trust level	1		2		3		4	
	B	Exp(B)	B	Exp(B)	B	Exp(B)	B	Exp(B)
Gender (female=0)	0.368	1.445	0.404	1.498	0.378**	1.460	-0.018	0.982
Age	-0.467***	0.627	-0.138	0.871	-0.371***	0.690	-0.128	0.880
Place of residence (rural=0)	0.281	1.324	-0.240	0.787	-0.097	0.908	-0.326*	0.722
Political affiliation	0.382	1.465	0.734**	2.083	0.099	1.104	0.051	1.053
Education	-0.183**	0.833	-0.071	0.932	-0.180***	0.836	-0.095	0.909
Income (lower=0)	0.151	1.163	-0.599*	0.202	-0.543	0.581	0.451	1.570
Occupation (public sector unit)								
Private enterprise	-0.316	0.729	0.622	1.863	0.586	1.797	0.429	1.536
Social organization	-0.701	0.496	-1.434	0.238	-0.014	0.986	-0.241	0.786
Others	-0.959	0.383	-0.301	0.740	0.646	1.908	-0.010	0.990
Family size								
Family size×education	0.010	1.010	-0.003	0.997	0.035**	1.035	0.015	1.015
Family size×income	-0.503	0.682	0.204	1.227	-0.584**	0.559	-0.511*	0.600
Family size×private enterprise	-0.020	0.980	0.069	1.071	-0.095	0.909	0.011	1.012
Family size×social organization	0.193	1.213	-0.149	0.861	-0.076	0.927	-0.171	0.843
Family size×others	0.310	1.364	0.208	1.232	0.162	1.176	0.110	1.116
Intercept	2.225*		0.523		3.053***		1.547	

Table S6-3: Multinomial regression results of moderating effects for institution trust

Institution trust-3								
Trust level	1		2		3		4	
	B	Exp(B)	B	Exp(B)	B	Exp(B)	B	Exp(B)
Gender (female=0)	0.329	1.389	0.618*	1.855	-0.009	0.991	-0.035	0.965
Age	-0.355**	0.701	-0.428**	0.652	-0.246*	0.782	-0.090	0.914
Place of residence (rural=0)	0.276	1.318	0.215	1.240	-0.092	0.912	-0.281	0.755
Political affiliation	0.263	1.301	-0.089	0.915	-0.097	0.907	-0.007	0.993
Education	0.178	1.195	0.107	1.113	0.167	1.182	0.145	1.156
Income (lower=0)	0.289	1.336	0.791	2.205	-0.825	0.438	-0.595	0.552
Occupation (public sector unit)								
Private enterprise	-1.359	0.257	0.832	2.298	0.185	1.203	1.094	2.987
Social organization	0.887	2.428	2.936	18.842	0.863	2.369	-0.002	0.998
Others	0.374	1.453	-0.717	0.488	0.615	1.850	0.302	1.353
Policy perception								
Policy perception×education	-0.063**	0.939	-0.026	0.974	-0.050*	0.951	-0.032	0.969
Policy perception×income	-0.912*	0.402	-1.242*	0.289	-0.591	0.554	-0.579	0.560
Policy perception×private enterprise	-0.152	0.859	0.118	1.125	-0.127	0.881	-0.051	0.950
Policy perception×social organization	0.386	1.471	-0.093	0.911	0.074	1.077	-0.339	0.712
Policy perception×others	-0.075	0.928	-0.834	0.434	-0.159	0.853	0.069	1.071
Intercept	3.251*		2.495		3.126*		2.752	
